# Acute Monoblastic Leukemia Presenting with Multiple Granulocytic Sarcoma Nodules

**DOI:** 10.4274/tjh.2015.0354

**Published:** 2017-03-01

**Authors:** Asude Kara, Aslı Akın Belli, Yelda Dere, Volkan Karakuş, Şükrü Kasap, Erdal Kurtoğlu, Mine Hekimgil

**Affiliations:** 1 Muğla Sıtkı Koçman University Training and Research Hospital, Department of Dermatology, Muğla, Turkey; 2 Muğla Sıtkı Koçman University Faculty of Medicine, Department of Pathology, Muğla, Turkey; 3 Muğla Sıtkı Koçman University Training and Research Hospital, Department of Hematology, Muğla, Turkey; 4 Muğla Sıtkı Koçman University Faculty of Medicine, Department of Plastic Surgery, Muğla, Turkey; 5 Antalya Training and Research Hospital, Clinic of Hematology, Antalya, Turkey; 6 Ege University Faculty of Medicine, Department of Pathology, İzmir, Turkey

**Keywords:** Granulocytic sarcoma, Acute monoblastic leukemia, CD34, Myeloperoxidase

A 76-year-old male presented to the department of plastic surgery with multiple nodules on his legs for 1 month. On examination, there were five discrete, violaceous nodules with a size of 0.5-3 cm on the legs (Figure 1). Laboratory tests revealed the following: white blood cell count of 3.6x10^9^/L, red blood cell count of 1.54x10^12^/L, platelet count of 82x10^9^/L, hemoglobin of 4.45 g/dL, and lactate dehydrogenase of 266 U/L. Due to pancytopenia, the patient was referred to the department of hematology before the excision. Peripheral blood smear showed 50% neutrophils, 40% lymphocytes, 8% monocytes, and 2% atypical cells. An excisional biopsy of skin lesions and a bone marrow biopsy (BMB) were performed. The BMB revealed monoblastic cell infiltration (40%) and immunohistochemical stains were positive with CD34 and myeloperoxidase (Figures 2A-2D). CD13, CD34, CD117, CD4, CD33, myeloperoxidase, CD38, and CD11c were detected in the blastic cells, which formed 31.4% of the population, by flow cytometry. The results were compatible with monoblastic leukemia and no genetic abnormalities were found. Histopathologically reactive lymphoplasmacytic infiltration in the dermis, including occasional blastic cells with morphologic features similar to the BMB findings like folded nuclei (Figures 3A-3D), was detected and diagnosed as granulocytic sarcoma (GS). However, the patient refused chemotherapy with azacitidine. Since cutaneous involvement of GS is rare and indicates poor prognosis, GS should be remembered in the differential diagnosis of suddenly emerging nodules and pustules [1,2].

## Figures and Tables

**Figure 1:**
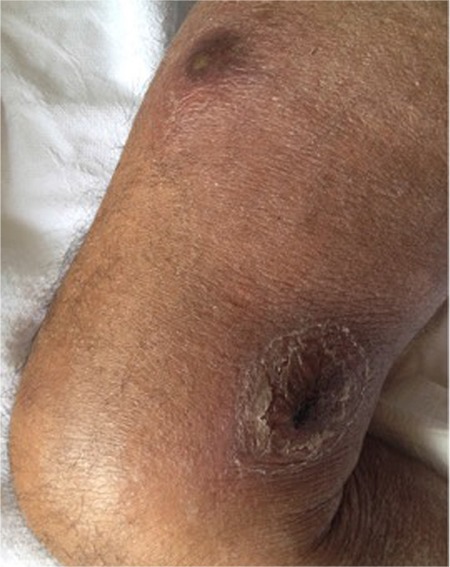
Violaceous nodules with central pustules and scaling on the right leg.

**Figure 2:**
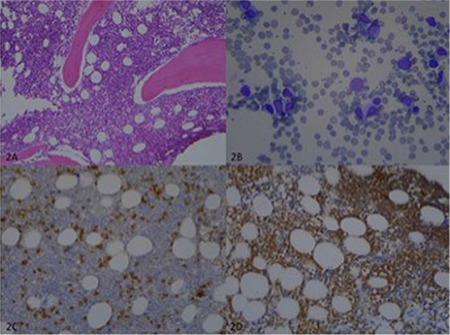
(A) Bone marrow biopsy showing hypercellularity (H&E, 100x). (B) Bone marrow aspiration smear showing erythroblasts and blastic cells with nuclear indentation (Giemsa, 400x). (C) CD34 (+) blastic cells (200x). (D) Myeloperoxidase (+) blastic cells (200x).

**Figure 3:**
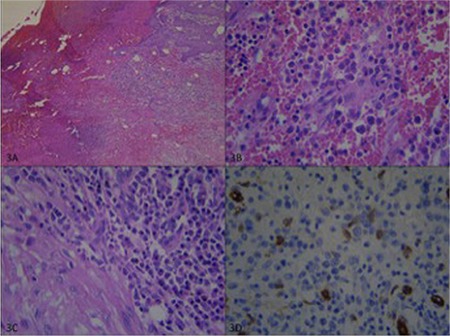
(A) Ulceration and pseudo-epitheliomatous hyperplasia in the epidermis, and inflammatory infiltration with capillary vessel proliferation under the epidermis (H&E, 40x). (B, C) Occasional blastic cells with folded nuclei that show monoblastic morphology similar to the bone marrow and plasma cells with thin-walled capillaries (H&E, 400x). (D) CD34 staining showing positivity in the endothelial cells intensely and scattered blasts (400x).
